# Assessing the Direct Binding of Ark-Like E3 RING Ligases to Ubiquitin and Its Implication on Their Protein Interaction Network

**DOI:** 10.3390/molecules25204787

**Published:** 2020-10-19

**Authors:** Dimitris G. Mintis, Anastasia Chasapi, Konstantinos Poulas, George Lagoumintzis, Christos T. Chasapis

**Affiliations:** 1 Laboratory of Statistical Thermodynamics and Macromolecules, Department of Chemical Engineering, University of Patras & FORTH/ICE-HT, 26504 Patras, Greece; dimitris.g.mintis@gmail.com; 2 Biological Computation & Process Lab, Chemical Process & Energy Resources Institute, Centre for Research & Technology Hellas, 57001 Thessaloniki, Greece; chasapi@certh.gr; 3 Laboratory of Molecular Biology and Immunology, Department of Pharmacy, University of Patras, 26504 Patras, Greece; kpoulas@upatras.gr; 4 Institute of Research and Innovation-IRIS, Patras Science Park SA, Stadiou, Platani, Rio, 26504 Patras, Greece; 5 NMR Center, Instrumental Analysis Laboratory, School of Natural Sciences, University of Patras, 26504 Patras, Greece; 6 Institute of Chemical Engineering Sciences, Foundation for Research and Technology, Hellas (FORTH/ICE-HT), 26504 Patras, Greece

**Keywords:** E3 RING ligases, ubiquitin, molecular dynamics, PPI network

## Abstract

The ubiquitin pathway required for most proteins’ targeted degradation involves three classes of enzymes: E1-activating enzyme, E2-conjugating enzyme, and E3-ligases. The human Ark2C is the single known E3 ligase that adopts an alternative, Ub-dependent mechanism for the activation of Ub transfer in the pathway. Its RING domain binds both E2-Ub and free Ub with high affinity, resulting in a catalytic active Ub_R_-RING-E2-Ub_D_ complex formation. We examined potential changes in the conformational plasticity of the Ark2C RING domain and its ligands in their complexed form within the ubiquitin pathway through molecular dynamics (MD). Three molecular mechanics force fields compared to previous NMR relaxation studies of RING domain of Arkadia were used for effective and accurate assessment of MDs. Our results suggest the Ark2C Ub-RING docking site has a substantial impact on maintaining the conformational rigidity of E2-E3 assembly, necessary for the E3’s catalytic activity. In the Ub_R_-RING-E2-Ub_D_ catalytic complex, the Ub_R_ molecule was found to have greater mobility than the other Ub, bound to E2. Furthermore, network-based bioinformatics helped us identify E3 RING ligase candidates which potentially exhibit similar structural modules as Ark2C, along with predicted substrates targeted by the Ub-binding RING Ark2C. Our findings could trigger a further exploration of related unrevealed functions of various other E3 RING ligases.

## 1. Introduction

**Figure 1:**
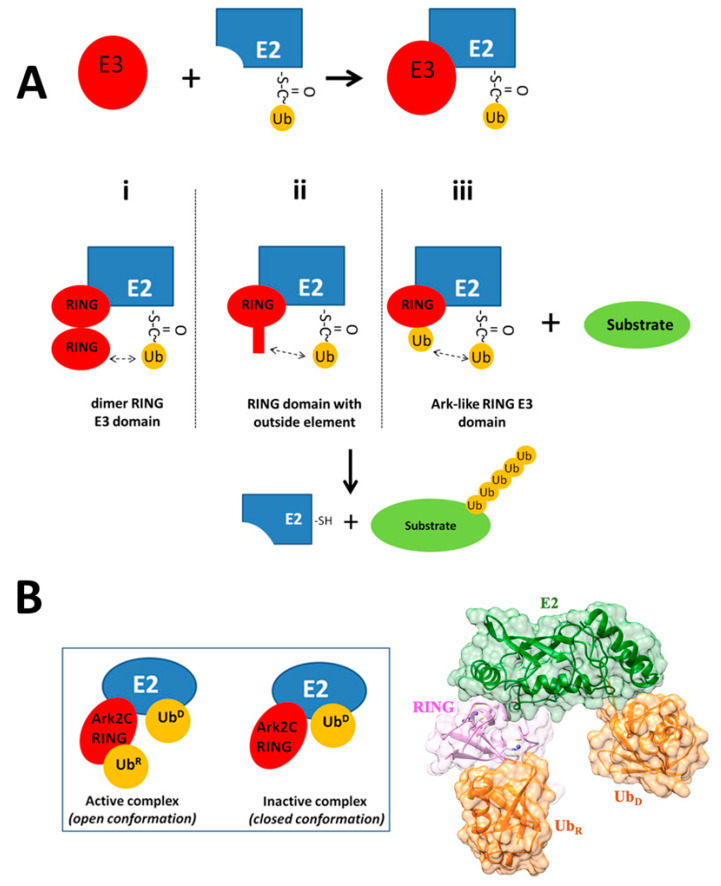
(**A**) Stabilization of the closed conformation of the E2-Ub thioester conjugation enzyme by E3 RING ligases. In the schematic interaction, E2 is shown to interact with: (**i**) Canonical dimeric E3 RING domain (e.g., cIAP2, RNF4), (**ii**) RING ligases with an additional *N*-terminal component (e.g., RNF38), (**iii**) Monomeric E3 RING ligases of the Arkadia type (e.g., RNF165). Arrows with dashed line signifies interactions favoring the activation of Ub for transfer to substrates (“closed conformation” or “E2~Ub”); (**B**) Active and inactive complexes (left panel) and 3D structure of active complex (right panel) of Ark2C with E2 enzyme and Ub.

The ubiquitin-proteasome system (UPS) in eukaryotes is an important system for the degradation of most short-lived proteins. The enzymatic ubiquitin (Ub) system is composed of Ub (a conserved 76-residue protein) and the E1 (ubiquitin-activating enzyme), E2 (ubiquitin-conjugating enzyme), and E3 ligases (Figure 1A) [1]. The system labels proteins for degradation by the UPS in three steps [2]. The first step is the transfer of Ub to E1 ligase fueled by the hydrolysis of ATP. In the second step, Ub is transferred to an E2 ligase connected via a thioester bond formed between an E2 Cys and a non-Cys (usually a Lys) residue of the substrate. Transfer of Ub from E2 to the target molecule is mediated by E3, which binds both E2 and substrate molecules to produce an isopeptide bond between the Ubs’ carboxyl group and substrates’ Lys residue [1].

Up to 1000 human E3 ligases have been described, dictating the substrate specificity of the much scarcer E1 and E2 [3,4]. The largest family of E3 ligases is the RING (Really Interesting New Gene) Zn finger proteins [5,6,7,8]. The RING domains of the E3 ligases are the specific molecular scaffolds that bring together E2 and the target proteins, allowing the transfer of Ub from E2 to the substrate (Figure 1A). To enhance the rate of Ub transfer, E3 ligases stabilize specifically the “closed conformation” of the E2-Ub thioester conjugation enzyme, thus activating the thioester bond (closed conformation) for the transfer of the Ub_D_ molecule (donor ubiquitin) to the substrate [9]. With this aim, the RING domain of E3 usually requires dimerization, where one RING subunit recruits E2- Ub_D_ via a canonical RING-E2 interface. At the same time, the other subunit establishes contacts with Ub_D_ (Figure 1Ai) so that the closed conformation of E2-Ub_D_ is favored [10,11,12].

RING domains can also be monomeric (Figure 1Aii). In this case, an additional component from the E3 ligase outside the RING domain (such as the phosphorylated linker tyrosine in Cbl E3 ligase [13], or a loop at the N-terminus of the RNF38 RING domain [14] makes sufficient contacts with Ub_D_, to populate the E2-Ub_D_ species in the closed active configuration. In an alternative mechanism for the activation of Ub_D_ transfer (known as “Ark-like”), a monomeric RING domain of the human RNF165 E3 ligase (Ark2C) may bind to E2-Ub_D_ and free Ub_R_ with high affinity. This results in the stabilization of the Ub_R_-RING-E2-Ub_D_ complex (open conformation; Figure 1B) through the development of new Ub_R_-Ub_D_ contacts (Figure 1Aiii), which prime Ub_D_ for catalysis and enhance ubiquitin transfer to the substrate [15]. Furthermore, a mutated Ark2C RING domain (M313A), which cannot bind free Ub_R_, has been co-crystallized with a mutated E2-Ub_D_ conjugate. The obtained RING-E2-Ub_D_ complex (in a closed conformation) (Figure 1B) is not catalytic, and the Ark2C is unable to discharge the Ub_D_ [15].

RING-type E3s are implicated as tumor suppressors, oncogenes, and mediators of endocytosis, and play critical roles in complex multi-step processes such as DNA repair and activation of NF-κB signaling. A RING-type E3 may have multiple substrates, and several E3s can target the same substrate. Not surprisingly, the mechanisms of substrate recognition by RING-type E3s are highly varied and occur in the context of networks of interactions that often also include homologous to the E6-associated protein carboxyl terminus (HECT) E3s and deubiquitinating enzymes (DUBs).

Arkadia (RNF111) is a 154 amino acid RING E3 enzyme that is differentially expressed in many cancer types, such as breast, pancreatic, and colon, and in some other diseases like Parkinson’s disease [16,17]. Arkadia interacts with various negative regulators of the TGF-β pathway, including Smad4, Smurf2, c-Ski, and SnoN, to up-regulate TGF-β signaling [17,18,19,20,21,22,23,24], and thus represents a possible therapeutic target [24,25]. It is also an essential component of the A20 ubiquitin-editing complex in the periphery and can negatively regulate NF-κB signaling in human monocytic cell lines [21]. Arkadia contains a RING-finger domain at its C-terminus required for ubiquitin-protein ligation. The E3 ubiquitin ligase function of Arkadia requires interaction with the E2 enzyme UbcH5B [26], and NMR studies of this interaction revealed a significant role for the RING-H2 domain in E2 recognition and binding [27,28,29,30]. The RING domain of Ark2C/RNF165 E3 ligase retains high sequence homology (85%) with Arkadia. Both exhibit similar ββα RING core topology, suggesting that these two proteins may promote ubiquitin transfer in a very similar manner [27,28]. Arkadia and Ark2C are the first reported E3 RING ligases with Ub-RING docking site [15].

In this work, we employed MD simulations to examine potential changes within the ubiquitin pathway on the conformational plasticity of the Ark2C RING domain and its ligands. Three different molecular mechanics force fields have been investigated to determine MDs’ efficiency and accuracy and compared to previous NMR relaxation studies of the Arkadia/RNF111 RING domain (homologous of Ark2C). Our results demonstrate that Ark2C Ub-RING docking site has a strong effect on maintaining the conformational rigidity of E2-E3 assembly, necessary for the E3’s catalytic activity. Moreover, in the Ub_R_-RING-E2-Ub_D_ catalytic complex, the Ub_R_ molecule was found to have greater mobility than the other E2-Ub. The in silico network-based analysis was also used to identify E3 RING ligase candidates potentially exhibiting similar structural modules as Arkadia2 and predicting substrates attacked by the Ub-binding RING Ark2C ligase. It seems that there are other E3s that could also bear similar structural characteristics in their RING domains. If proven, this could assign them new features and implicate them in several other cellular functions that are as yet unrevealed.

## 2. Results

### 2.1. Structural Fluctuations in Ark2C RING Domain, after Binding to Ligands

**Figure 2:**
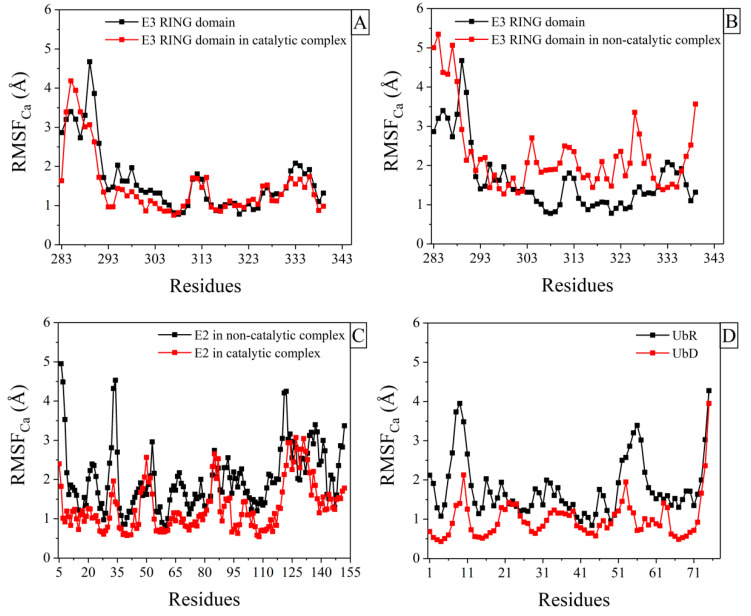
(**A**) Structural fluctuations (RMSF of Ca atoms per residue) for the core of RING domain of the human Ark2C E3 ligase simulations (black) and in the complex with E2-Ub in the catalytic conformation (Ub–RING-E2-Ub, red); (**B**) Structural fluctuations (RMSF of Ca atoms per residue) for the core of RING domain of the human Ark2C E3 ligase simulations (black) and in the complex with E2-Ub in the non-catalytic conformation (RING-E2-Ub, red); (**C**) Structural fluctuations (RMSF of Ca atoms per residue) for the E2 enzyme in the non-catalytic conformation of the E3-E2-Ub_D_ complex (black) and in the catalytic conformation of the Ub_R_-E3-E2-Ub_D_ complex (red); (**D**) Structural fluctuations (RMSF of Ca atoms per residue) for the Ub_R_ bound to RING domain (black), and Ub_D_ bound to E2 (red) in the catalytic conformation of the Ub_R_-E3-E2-Ub_D_ complex.

The overall plasticity of the core of Ark2C RING polypeptide was slightly decreased (Figure 2A) in the active catalytic complex (Figure 1B, Ub_R_-RING-E2-Ub_D_, PDB ID: 5D0K [15]). Residues located in the region between Ser290 and His317, associated with the binding interfaces between RING domain and E2 and Ub_R_, based on the X-ray structure of the catalytic complex, exhibited remarkably decreased structural fluctuations (RMSF measures). MD simulations were also performed for the mutated Ark2C RING domain (M313A) when it is complexed with the mutated E2 (C85K and S22R) bound to Ub_D_. This complex (Figure 1B, RING-E2-Ub_D_, PDB ID: 5D0Μ) was found to be non-catalytic. The inability of Ub transfer of this complex (which was crystallized in closed conformation) was explained by the absence of bound Ub to the RING domain and the different relative orientation of the mutated RING-E2 complex compared to the published RING-E2 structures [15]. According to our results, the whole RING polypeptide exhibited increased mobility in this complex (a significant increase of RMSF measures, Figure 2B). The comparison of the order parameter, S2, of the NH bonds of the Ark2C RING domain in its catalytic complex (Ub_R_-RING-E2-Ub_D_) with the mutated RING domain in the non-catalytic complex (RING-E2-Ub_D_) is provided in Figure S11A. A remarkable consistency is observed with conclusions derived from the RMSFs calculations.

### 2.2. Structural Fluctuations in E2 Enzyme and Ubs after Binding to Ligands

Comparison of RMSFs in E2 enzyme between the catalytic assembly (Ub_R_-RING-E2-Ub_D_) and the non-catalytic (RING-E2-Ub_D_) revealed that in the second complex E2 exhibited increased mobility (a significant increase of RMSF measures, Figure 2C). Furthermore, calculations of RMSFs were carried out for the Ubs in the active complex Ub_R_–RING-E2-Ub_D_ (PDB ID: 5D0K [15]), revealing that the Ub_R_ bound to RING domain had greater mobility (Figure 2D). These observations regarding the increase of E2 and Ub_R_ mobility were also consistent with calculations obtained from the order parameter (derived from a 5ns time-window) of the NH bonds for E2 enzyme and Ub_R,_ respectively (Figure S11B,C). Notably, the order parameter for ubiquitin, as obtained from previous experiments [31,32], is in perfect agreement with observations made in this study for the Ub_D_ (Figure S11C).

### 2.3. Identification of Putative Ub-Binding E3 RING Ligases

Among all E3 RING ligases, Arkadia and Ark2C are the first ones reported to have a Ub-RING docking site. Consequently, we performed in silico, network-based experiments to determine other E3 RING ligases that may exhibit similar attributes. The analysis was based on the assumption that interacting proteins are not necessarily similar to each other and, therefore, identifying putative interacting partners based on network similarity might not be sufficient. Instead, one can expect proteins to interact if one of them is similar to the other’s partners. This approach was recently presented in [33], and it mathematically relies on network paths of length = 3. Proteins are expected to interact if linked by multiple ℓ = 3 paths in the human interactome. In our work, we used this strategy both for the identification of putative Ub-binding E3 RING ligases, described here, as well as the identification of potential Arkadia and Ark2C interactors (see Section 2.4).

**Figure 3:**
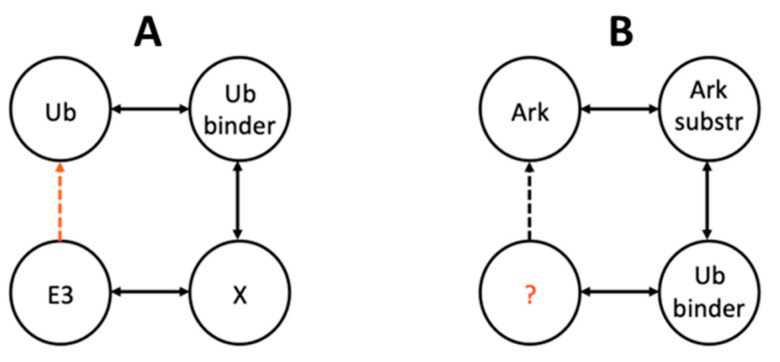
Illustration of the ℓ = 3 network-based approach used to identify (**A**) potential E3 RING ligases with Ub docking sites and (**B**) putative Ark and Ark2C interactors. In each case, the element under question is presented in red.

In the case of putative Ub-binding E3 RING ligases, we applied the network analysis of ℓ = 3 as follows: The first path links Ub to known Ub-binding proteins, the second path links Ub-binding proteins to other known interactors, and the third path represents the E3 RING ligases that have a known connection to the abovementioned interactors. These E3 ligases are expected to be structurally similar to Ub-binding proteins and, therefore, potentially bind to Ub (Figure 3A).

A detailed list of Ub-binding proteins and their known interactors, as outlined in the Methods Section, as well as E3 RING ligases and their known substrates, was developed. Substrates of 244 E3 RING ligases (out of 331) were found to be common with interactors of Ub-binding proteins (see Supplementary Material S2), implying that they might share structural features that permit this interaction. The candidate list was subsequently filtered based on the assumption that a multitude of common interactors (i.e., higher connectivity degree) between Ub-binding proteins and an E3 RING ligase increase the latter’s likelihood also displays a Ubiquitin binding site. The best candidates that resulted from the analysis are presented in Table 1.

KEGG enrichment analysis of the putative Ub-binding E3 ligases revealed that the most representative pathways were related to cancer (23.5%), viral infection (10.2%), bacterial infection (9%), and ubiquitin-mediated proteolysis (8.2%).

### 2.4. Network Analysis Reveals Potential Arkadia and Ark2C Interactors

Putative interactors for Arkadia and Ark2C were identified based on their connection to other Ub binding proteins through common interactors. All known substrates were mined for each Arkadia protein and compared to the complete dataset of known Ub-binding protein interactors. A sorted list was subsequently produced, revealing the Ub-binding proteins that share most interactors with each Arkadia. Higher connectivity (most shared interactors) implies structural similarity. Thus, other interactors of the top-rated Ub-binding proteins could also potentially interact with Arkadia. A list of the most connected Ub-binding proteins for each Arkadia protein can be seen in Table 2. The complete dataset is available as Supplementary Material (S3). The majority of reoccurring Ub-binding protein interactors constitute the putative Arkadia interactors.

The network analysis of ℓ = 3, in this case, was formed as follows: the first edge links Arkadia or Ark2C to their known substrates, the second edge links these substrates to their interactors that are also Ub-binding proteins, and the third edge connects these Ub-binding proteins to other proteins with which they interact. These proteins can potentially bind to Arkadia or Ark2C, respectively (Figure 3B).

Two of three potential interactors of Arkadia are the 40S ribosomal protein S27a and the 60S ribosomal protein L40 [34], which are both synthesized as a fusion protein with ubiquitin. The third is the inhibitor of nuclear factor kappa-B kinase subunit beta, a serine kinase [35] that plays an essential role in the NF-κB signaling pathway. The remaining two identifiers (P0CG48 and P0CG47) belong to Ubiquitins, which does not contradict the analysis strategy as Ubiquitins bind to Arkadia but do not provide insights for possible interactions. The majority of the potential interactors of Ark2C includes enzymes like Ubiquitin-conjugating enzymes E2s (UBE2D2 and UbcH6), Superoxide dismutase [Cu-Zn], transferases such as E3 ubiquitin-protein ligase parkin (O60260), components of Cullin-RING E3 ubiquitin ligases complexes (Cullin-3; Q13618), Histone acetyltransferase KAT5 (Q92993), protein tyrosine kinase (JAK2; O60674) and it is receptor (Epidermal growth factor receptor; P00533).

## 3. Discussion

Ark2C RING E3 ligase has a unique structural feature in binding both to the E2-Ub_D_ thioester conjugate and free Ub_R_ molecule, resulting in the formation of the Ub_R_-RING-E2-Ub_D_ complex, which enhances ubiquitin transfer to a substrate. In this study, detailed fully atomistic MD simulations were employed to detect changes in the conformational plasticity of Ark2C RING domain and its ligands in their complexed form. In the active catalytic complex of the Ark2C RING domain bound to both Ub_R_ and E2-Ub_D_ (Figure 1B), the E3 RING ligase scaffold acquired light decreased flexibility (Figure 2A). On the other hand, the major part of the mutated Ark2C RINGM313A domain had increased mobility in the inactive complex (RING-E2-Ub_D_, PDB ID: 5D0Μ, Figure 1B). Thus, the increased mobility of the mutated Ark2C RING domain in its assembly may be essential for the reported limited ability to promote ubiquitin transfer [15]. The structure of the inactive complex of Ark2C was described by Wright et al. [15] as the conjugate conformation, just before catalytic priming, perhaps because the essential Ub_R_ is missing from the complex. Based on the current results, the additional Ub_R_, when bound to RING domain in the assembly (Ub_R_-RING-E2-Ub_D_), stabilizes the conformation of the E2-E3 assembly (significant and slightly reduced RMSF measures for E3 and E2 enzymes respectively, Figure 2B,C). This Ub_R_-induced rigidity of E2-E3 active complex probably enhances the nucleophilic attack of the thioester bond in the E2-Ub_D_ conjugate.

Furthermore, the binding of Ub_R_ to RING domain causes allosterically decreased RMSF measures for the amino acids of helix α2 (Gln318-Leu325). In the C-part of the helix is located the highly conserved RING Trp324 (or Trp972 for Arkadia) [6]. Because it is well known that this Tryptophan is essential for the recruitment of E2 and its interaction with the monomeric Arkadia RING domain [6], the Ub-stimulated stabilization of this region is likely to be necessary for the enzymatic activity of Ark2C. It should be emphasized that in the catalytic complex of Ark2C (Ub_R_-RING-E2-Ub_D_), the Ub_R_ molecule had greater mobility than Ub_D_ (bound to E2) (Figure 2D). The increased mobility of Ub_R_ element in the assembly may be necessary for the catalytic activity. This Ub_R_ could act as a flexible tail that can facilitate the formation of Ub_R_-Ub_D_ contacts.

E3 RING ligases may regulate the cell cycle, apoptosis, gene transcription, cell signaling, and DNA repair critical for the onset and development of colorectal cancer and other types of cancer [17,20,23,24,36]. E3 RING ligases may also be involved in immune responses to pathogen infection [37], the recognition of methylated DNA by transcription factors [38], and Parkinson’s disease [39]. Additionally, E3 RING ligases provide scaffolds for the interaction of proteins involved in various vital pathways, including Alzheimer’s disease, MAPK signaling, NF-κΒ signaling, etc. Interestingly, gene ontologies of the putative Ub-binding E3 RINGs revealed by our network analysis suggest that their majority is involved in cancer and viral and bacterial infection pathways. We therefore strongly suggest that future biochemical studies must be conducted first to determine whether the predicted E3 RING ligases bind Ub molecules and second if the Ub-E3 docking site is located in their RING domain.

Although the identification of E2 enzymes as Arkadia and Ark2C interactors is an expected result, it is important since it confirms the reliability of the network-based analysis demonstrated herein. The prediction of new interactions for both Arkadia E3s could suggest possible participation in new biological processes, from the already reported targeting the ubiquitination of TGF-β signaling regulators (SMAD7 and SKI). For instance, the majority of the predicted interactors are involved in the initiation of the DNA damage response to double-strand breaks, metabolic processes (cellular, primary nitrogen compounds, and organic substances), signal transduction, cellular developmental processes (aging, differentiation, development), response to a stimulus (chemical, stress, abiotic and endogenous stimulus) and immune system processes (e.g., leukocyte migration, and activation, inflammation, etc.).

Structural bioinformatics combined with Protein–Protein Interaction (PPI) network analysis help to generate a qualitative understanding of how a biomolecule or drug works [40]. Perhaps even more importantly, integrative bioinformatics can generate hypotheses that lead to new experimental work. Thus, we studied this unique mechanism of Ark2 both at atomic and macroscopic levels. That is, we investigate whether the binding of a Ub molecule in the RING domain of ArK2C causes favorable changes in the dynamics of the E2-E3 complex for its stabilization. We also worked on the potential effects of this binding on Ark’s interactome network, allowing it to interact with new proteins (i.e., potential substrates) so its possible involvement in new biological pathways.

Our findings could prompt other researchers and us to investigate experimentally the potentials raised by the identification of novel Ub binding E3 RING ligases along with their interactors that could contribute to several human diseases.

## 4. Materials and Methods

### 4.1. Molecular Dynamics

All MD simulations conducted in this work were performed using GROMACS software (version 2016.3, free, open-source software, developed and maintained by the GROMACS development teams at the KTH Royal Institute of Technology, Stockholm, Sweden and Uppsala University, Upsala, Sweden) [41]. A description of the systems simulated is presented in Table 3, whereas simulation details are discussed in the Supplementary Material (S1).

#### Force Field Selection

To choose the appropriate molecular mechanics force field to study Ark2C RING domain and its ligand, the capability of three different force fields (AMBER-03 [42], AMBER99SB-ILDN [43], and AMBER99SB-STAR-ILDNP [44]) to accurately predict the RING domain of Arkadia/RNF111 as compared against previous NMR relaxation studies of this RING domain [27] was examined. A thorough and systematic comparison of the MD predictions regarding the root-mean-square deviation (RMSD), secondary structure, solvation, and order parameter, *S*^2^, of the NH bonds (on the backbone chain), as obtained from the three force fields and the previous NMR relaxation study [27], was undertaken in the present study. A brief description for calculating these properties from the MD simulations/trajectories is given in Section 2 of the Supplementary Material (S1).

From the calculation of the RMSD of the backbone atoms of the Arkadia RING_927–994_ domain (see Figure S1A), it was revealed that all three force fields compare considerably well, within statistical error, with the experimental value. On the contrary, from the calculation of the RMSD of the heavy atoms of the Arkadia RING_927–994_ domain (see Figure S1B), it was observed that although the AMBER-03 and the AMBER-99SB-STAR-ILDNP force fields were in good agreement, within the margin of the statistical error with the experimental value, AMBER-99SB-ILDN force field failed to do so. Notably, the time evolution of the RMSD and the time evolution of the radius of gyration and end-to-end distance of the Arkadia RING_927–994_ domain were also presented in Figures S2 and S3 of the Supplementary Material (S1), respectively, to ensure adequate convergence and confirm that adequate sampling was performed in the MD simulations.

Although the RMSD is commonly used to validate computational models against experiments, it should be taken with considerable care since the RMSD is dominated by considerable large errors. Based on this fact, in this work, we further examined the efficiency and accuracy of the three different force fields by comparing their predictions of the secondary structure and the hydration behavior of the Arkadia RING_927–994_ domain against experimental observables [27]. The AMBER-03 force field (see Figure S6) and the AMBER-99SB-ILDN force field (see Figure S7) failed to reproduce the β-sheet between residues Asp937-Glu939 and Val955-Arg957, respectively, whereas the AMBER-99SB-STAR-ILDNP force field (see Figure S5) predicted with remarkable stability (in respect to time) the β-sheet as well as the *α*-helix of the Arkadia RING_927–994_ domain as compared against the previous NMR study [27]. Furthermore, the solvation behavior of the Arkadia RING_927–994_ domain was assessed by computing the solvent accessible surface area (SASA). As can be seen in Table S1 of the Supplementary Material (S1), the AMBER-99SB-STAR-ILDNP force field was found to be in good agreement with the previous NMR study [27]. On the other hand, the AMBER-03 and the AMBER-99SB-ILDN force fields did not show the same consistency.

**Figure 4:**
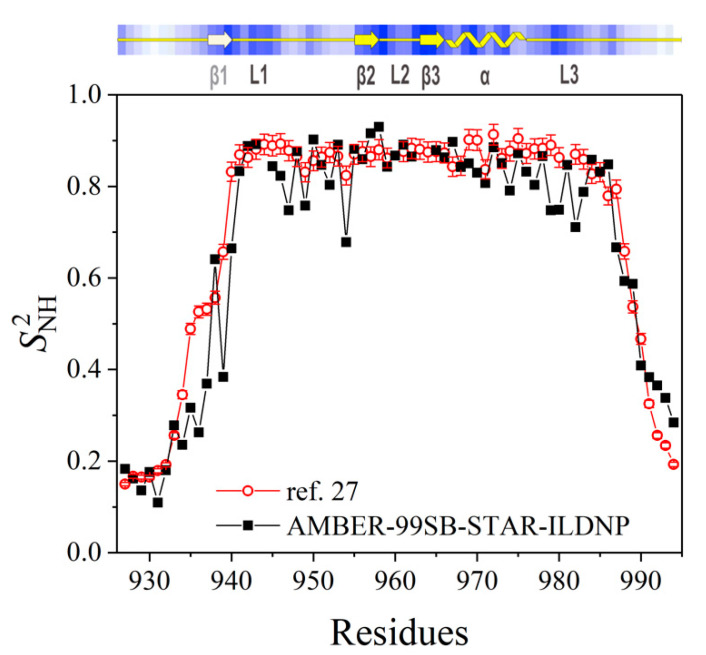
Comparison between the order parameter of the NH bond of the Arkadia RING_927–994_ domain predicted by employing the AMBER-99SB-STAR-ILDNP molecular mechanic’s force field and NMR relaxation data [27]. The top plane indicates observations regarding the secondary structure as obtained from the previous NMR-based study [27].

Lastly, the order parameter, *S*^2^, of the NH bonds (on the backbone chain) was computed using the three different force fields and compared against experimental findings obtained from the previous NMR relaxation study [27]. The order parameter, *S*^2^, was calculated from the MD simulation using Equation 6 (as shown in Section 2 of the Supplementary Material S1) derived from the 5ns time-window (time-window was selected carefully considering very well converged correlation functions). As can be seen in Figure 4, the AMBER-99SB-STAR-ILDNP force field was found to be in excellent agreement with experimental NMR data [27]. On the contrary, the AMBER-03 force field (see Figure S10A), although seemed to compare considerably well with observations obtained from the experiment it predicted a stiffer conformation for the residues 927–939 of the Arkadia RING927–994 and the AMBER-99SB-ILDN force field (see Figure S10B) predicted a stiffer conformation for residues 927–934 and 986–994, respectively. From this systematic investigation, we observed that the AMBER-99SB-STAR-ILDNP force field was capable of efficiently and accurately predicting the behavior of the RING domain of Arkadia/RNF111. Thus, it was decided to adopt the AMBER-99SB-STAR-ILDNP force field in this work for studying Ark2C RING domain and its ligand.

Notably, the MD simulations were repeated four times (for 150 ns each) to verify the AMBER-99SB-STAR-ILDNP force field accuracy. However, one should be aware that current classical force fields are known to be problematic in capturing the correct behavior of heavy metal ions, and also, it is acknowledged that the AMBER-99SB-STAR-ILDN force field over-stabilizes the secondary structure elements [45,46,47,48]. Although these issues are indeed a key part in capturing the accurate underlying microscopic picture of the Ark2C RING domain and its ligand studied here were not discussed or highlighted further in this work as they were beyond the scope of this current study.

### 4.2. Network-Based Analysis

The E3 RING ligase dataset, comprising 331 ligases, was extracted from a recently published E3 ubiquitin ligase collection [49] employing a comprehensive list of relevant resources such as Cell Signaling Inc., hUbiqutome, Qiagen Ubiquitin Ligases PCR Array, UbiProt Database, and DUDE v.1.0. For the E3 RING ligase substrate dataset, we chose a manually curated collection, which was used to evaluate the UbiBrowser platform [50]. This set was constructed by proven interactions through low-throughput methods. Even though it only contains substrates for a subset of our E3 RING ligase dataset, we opted to have a high-quality dataset (experimentally supported) rather than a dataset of predicted interactors given the known difficulty in E3 ligase substrate identification [51]. The Ub binding protein dataset was mined from the iUUCD database (version 2.0) [52], and their interactors were mined from the BioGRID web resource using the appropriate queries [53]. The identification of E3 RING ligases with potential Ub-binding capacity and the prediction of substrates targeted by the Ub-binding RING Arkadia2 ligase were performed using correlation strategies [54], inspired by the recently reported network-based methodology by Kovács et al. [33]. All constructed datasets can be found as Supplementary Material (S4).

### 4.3. Functional Annotation, Subcellular Localization and Protein Structure Visualization

Functional annotation of human targets was performed using the DAVID bioinformatics resource [55]. Current results are based on the human genome ontology analysis (including the human genome’s size and the various gene ontology terms) as the human UniProt IDs were used in the DAVID resource. Subcellular localization was predicted on the information provided by the UniProt catalog. CSF Chimera software (1.14, developed by the Resource for Biocomputing, Visualization, and Informatics at the University of California, San Francisco, CA, USA, with support from NIH P41-GM103311), was used to visualize all protein structures [56].

## 5. Conclusions

Integrative bioinformatics, such as structural and network-based systems biology, is a powerful technique that can influence experimental work by exploring molecular properties that are difficult or impossible to access wet-lab experiments. Herein, MD simulations were employed to investigate changes in the plasticity of key proteins that activate the ubiquitin-proteasome pathway through their participation in complexes. RMSF measures suggested changes of plasticity/rigidity in specific stretches after the formation of the complexes. Specifically, the Ark2C Ub-RING docking site has a significant impact on maintaining the conformational rigidity of E2-E3 assembly, necessary for the E3’s catalytic activity. In the Ub_R_-RING-E2-Ub_D_ catalytic complex, the Ub_R_ molecule was found to have greater mobility than the other E2-Ub (Ub_D_). PPI network-based analysis revealed that other than Ark2C E3 ligases could also bear similar structural characteristics in their RING domains. If this is proven, it could pave the way to identify other critical cellular functions of RING E3s, other than those known up to now, highlighting the current need for further studies in this field that would ultimately trigger productive future experimentation.

## Figures and Tables

**Table 1:** Top Ub-binding E3 RING ligase candidates based on network analysis involving their shared interactors with known Ub-binding proteins. The connectivity degree reflects the number of common interactors among Ub-binding proteins and E3 RING ligases and is indicative of the potential structural similarity of the E3 ligase to Ub-binding protein.

E3 RING Ligase (UniProt ID)	Protein Name	Connectivity Degree with Ub-Binding Proteins
P22681	E3 ubiquitin-protein ligase C.B.L.	35
Q00987	E3 ubiquitin-protein ligase Mdm2	31
O60260	E3 ubiquitin-protein ligase parkin	24
Q8IUQ4	E3 ubiquitin-protein ligase SIAH1	18
Q86TM6	E3 ubiquitin-protein ligase synoviolin	15
Q9Y4K3	TNF receptor-associated factor 6	14
O43255	E3 ubiquitin-protein ligase SIAH2	13
P38398	Breast cancer type 1 susceptibility protein	13
P35226	Polycomb complex protein BMI-1	12
Q13490	Baculoviral IAP repeat-containing protein 2	11

**Table 2:** Ub-binding proteins sharing the most common interactors (mentioned as connectivity degree) with Ark2C and Arkadia, respectively. The most frequently reccurring Ub-binding protein interactors constitute the putative Arkadia interactors. These were further refined, based on subcellular localization evidence.

	Ub-Binding Protein (UniProt ID)	Connectivity Degree with Arkadia	Potential Arkadia Interactors
**Ark2C**	Q96A37	7	O60260, O60674, P00441, P00533, P0CG47, P0CG48, P51965, P60709, P62837, Q07666, Q13501, Q13618, Q92993
	Q8IYW5	6	
	Q9UBN7	5	
	Q8TF42	5	
	P42566	5	
**Arkadia**	P03372	2	P0CG48, P0CG47, P62979, P62987, O14920

**Table 3:** A list of all systems simulated in this work with their relevant information.

No	Protein Complex Description		PDB ID	Force Field	Size of Cubic Box, x (Å)	Simulation Time (ns)
1	Arkadia E3 Ligase (RNF111)	SOLUTION NMR	2KIZ	AMBER-03	60	150
2				AMBER-99SB-ILDN-2010	80	150
3				AMBER-99SB-STAR-ILDN-2014	80	150
4	Ark2C E3 Ligase (RNF165)	X-RAY DIFFRACTION 2.65 Å	5D0I	AMBER-99SB-STAR-ILDN-2014	80	150
5	E3-E2-Ub non-catalytic complex	X-RAY DIFFRACTION 1.91 Å	5D0M	AMBER-99SB-STAR-ILDN-2014	92	150
6	Ub-E3-E2-Ub catalytic complex	X-RAY DIFFRACTION 2.65 Å	5D0K	AMBER-99SB-STAR-ILDN-2014	100	150

## Supplementary Materials

The following are available online: S1: Supplementary Material doc file that contains all the supplementary figures and tables; S2–S4: Supplementary Material with three excel files with supplementary data.
